# Ventilatory power, a cardiopulmonary exercise testing parameter for the prediction of pulmonary hypertension at right heart catheterization

**DOI:** 10.1016/j.ijcha.2020.100513

**Published:** 2020-04-21

**Authors:** Michele Correale, Ilenia Monaco, Armando Ferraretti, Lucia Tricarico, Monica Sicuranza, Anna Maria Gallotta, Ennio Sascia Formica, Gianfranco Acanfora, Matteo Di Biase, Natale Daniele Brunetti

**Affiliations:** a Cardiology Department, Ospedali Riuniti University Hospital, Foggia, Italy; b Department of Medical & Surgical Sciences, University of Foggia, Foggia, Italy

**Keywords:** Ventilatory power, Diastolic pressure gradient, Transpulmonary pressure gradient, Pulmonary vascular resistance, Cardiopulmonary exercise test, BMI, body mass index, CI, cardiac index, COPD, chronic obstructive pulmonary disease, Cpc-PH, combined post-capillary and pre-capillary pulmonary hypertension, CPET, cardiopulmonary exercise testing, DPG, diastolic pressure gradient (diastolic PAP – mean PAWP), ECG, electrocardiogram, EF, ejection fraction, Ipc-PH, isolated post-capillary pulmonary hypertension, 6MWT, 6-minute walking test, NYHA, New York Heart Association, PAH, pulmonary arterial hypertension, mPAP, mean pulmonary arterial pressure, PAsP, systolic pulmonary arterial pressure, PAWP, pulmonary artery wedge pressure, PetCO_2_, end-tidal carbon dioxide tension, Peak VO_2_, peak oxygen consumption, PH, pulmonary hypertension, PVR, pulmonary vascular resistance, RAP, right atrial pressure, RHC, right heart catheterization, RV, right ventricle, TPG, transpulmonary pressure gradient (mean PAP – mean PAWP), VE, ventilation, VE/VCO_2_, minute ventilation- carbondioxide production ratio, VP, ventilatory power

## Abstract

**Background:**

Several cardiopulmonary exercise test (CPET) parameters (peak VO_2_, PetCO_2_ and VE/VCO_2_) emerged as tools for the prediction of pulmonary arterial hypertension (PAH). Less is known on ventilatory power (VP) in patients with suspect PAH.

**Aim:**

To ascertain possible correlations between VP derived at CPET and hemodynamic parameters at right heart catheterization (RHC) indicative of PH.

**Methods:**

Forty-seven consecutive outpatients with suspect of PAH were assessed by CPET and RHC; VP was defined as peak SBP divided by the minute ventilation-CO2 production slope at CPET and Diastolic Pressure Gradient (DPG), Trans-pulmonary Pressure Gradient (TPG), mean pulmonary artery pressure (mPAP) and pulmonary vascular resistance (PVR) at RHC were also assessed and compared with VP.

**Results:**

VP values were inversely related to mPAP (r −0.427, p 0.003), DPG (r −0.36, p 0.019), TPG (r: −0.43, p 0.004), and PVR (r −0.52, p 0.001). Correlations remained significant even after correction at multivariate analysis for age and gender. VP values below median identified subjects with mPAP ≥ 25 mmHg with an odds ratio of 4.5 (95% confidence interval 1.05–19.36, p < 0.05), an accuracy of 0.712 at ROC curve analysis (95% confidence interval 0.534–0.852, p < 0.05) and a positive predictive power 82%.

**Conclusions:**

In patients with suspected PAH, VP assessed at CPET might provide further information in predicting PAH at RHC. Correlations with PVR and DPG may be helpful in differentiating patients with isolated post-capillary PH from those with combined post-capillary and pre-capillary.

## 1 Background

Pulmonary hypertension (PH) is a clinical disorder characterized by an increased pulmonary arterial pressure due to several clinical conditions, including cardiovascular and respiratory disorders. Mean pulmonary arterial pressure (PAP) measured at right heart catheterization (RHC) ≥ 25 mmHg is required for a definitive diagnosis of PH [1]. According to international guidelines, patients who have symptoms, signs and history suggestive for PH should undergo a multi-step work-up, which includes non-invasive tests such as electrocardiogram, echocardiography, pulmonary function test, high resolution computed tomography, and ventilation/perfusion lung scan before RHC, as invasive test. Although the most widely used exercise test in PH specialized centers is the 6-minute walking test (6MWT), this test provides only limited clinical information. Cardio-pulmonary exercise test (CPET), instead, may provide both further prognostic information in PH patients and a comprehensive pathophysiological evaluation of exercise limitation and dyspnea [2].

Recently, ventilatory power (VP), a new CPET index defined as the peak systolic blood pressure divided by VE/VCO2 slope, combining physiology inherent in the VE/VCO2 slope to peripheral pressure, was used to evaluate the response of sequential combination therapy in patients with PAH [3]. Used for the first time by Forman et al. [4] in patients with heart failure, VP was in this study one of the strongest indicator of prognosis. VP was also used as screening tool in assessing the functional significance of coronary disease and exercise tolerance [5].

Aim of this study was therefore to ascertain possible correlations between VP derived by CPET and hemodynamic parameters at RHC indicative of PH.

## 2 Methods

Between 1st March 2010 and 1st March 2018, a total of 47 consecutive outpatients with suspected of PAH based on non-invasive findings of increased PAsP or additional echocardiographic features at echo-color-Doppler assessment (increased dimensions of RH chambers, abnormal shape and function of the interventricular septum, increased right ventricular (RV) wall thickness, and dilated main pulmonary artery) and able to perform an CPET were enrolled in the study.

Patients underwent pulmonary and cardiology assessment by clinical exam, and assessed by CPET and RHC as reported elsewhere [4], [6], [7]. Medical history, heart rate, systolic blood pressure, Body Mass Index, NYHA class, and medications were recorded.

All patients gave an informed consent. The study was approved by local ethical committee and was held according the ethical standards for experiments in human subjects established by the Declaration of Helsinki.

### 2.1 Cardiopulmonary exercise testing (CPET)

Incremental CPET was performed on a *cyclo*-ergometer (Ergometrics Lode Medical Tecnology-Corival, Groningen, The Netherlands) using a ramp protocol that was personalized with the objective of each patient reaching a maximum exercise within 8 to 10 min. After 60 s of unloaded pedaling at 60 revolutions per minute, work was continuously increased at a rate of 4–10 W/min starting at 0 W. In all cases, breath-by-breath expiratory gases and ventilation analysis were performed (Vmax Spectra 29S, Sensor Medics, Yorba Linda, CA). AT was measured with the V-slope analysis from the plot of VCO_2_ vs. VO_2_ on equal scales. The AT value was confirmed by ventilatory equivalents and end-tidal pressures of CO_2_ and O_2_. If no agreement was obtained, the AT was considered not identified. The VO_2_/work rate relationship was evaluated throughout the entire exercise. The VE/VCO_2_ slope was calculated as the slope of the linear relationship between VE and VCO_2_ from 1 min after the beginning of loaded exercise to the end of the isocapnic buffering period. Peak exercise ventilation as % of a predicted value (VE%) was also reported. VP was defined as peak systolic blood pressure divided by the minute ventilation-CO2 production. ECG, blood pressure and heart rate were assessed continuously.

### 2.2 Right heart catheterization (RHC)

Hemodynamic assessment was performed by RHC (using a Swan-Ganz pulmonary artery catheter, CCOmbo V, Edwards Lifesciences, Irvine, CA, USA). Pulmonary arterial (systolic, diastolic and mean), right atrial, and pulmonary capillary wedge pressures (PWP) were recorded at the end of a quiet respiratory cycle. Oxygen saturations in the superior vena cava, inferior vena cava, pulmonary artery, and femoral artery were obtained. Pulmonary vein saturation was assumed at 98%. Pulmonary and systemic flows were obtained by the Fick principle using table-derived oxygen consumption values and calculated oxygen content at the correspondent different sites. The *trans*-pulmonary pressure gradient was defined as the difference between the mean pulmonary arterial pressure and the mean pulmonary capillary wedge. Therefore the DPG (defined as diastolic PAP - mean PAWP) has been reported. Pulmonary and systemic vascular resistance indices were calculated using the standard formula. A PAWP > 15 mmHg excluded the diagnosis of pre-capillary PAH.

### 2.3 Statistical analysis

Continuous variables were expressed as mean ± standard deviation and compared with Student’s *t*-test or Mann-Whitney *U* test as required, categorical variables as percentages and compared with χ2 or Fisher test as required. Normal distribution was analyzed with Kolmogorov-Smirnov & Lilliefors test for normality.

Linear correlations were determined by measuring the Pearson’s correlation coefficient: univariate results were corrected in a multivariate analysis for age and gender.

Logistic regression was used to calculate odd ratio (OR) with 95% confidence interval. Receiver operating characteristics curve analysis was used to assess the area under the curve. A p < 0.05 was considered as statistically significant.

## 3 Results

Forty-seven consecutive outpatients (61 ± 11 years, 53% male) matching the inclusion criteria were enrolled in the study. Their clinical characteristics are given in Table 1. Hemodynamic parameters at RHC and CPET are given in Table 2.

**Table 1:** Population’s characteristics.

Clinical parameters	Mean ± SD	Percentage
Age (years)	61 ± 11	
Male %		53%
III-IV WHO FC class		62.5%
PAsP (mmHg)	63.4 ± 21.8	
PH (all groups) %		65.9%
PH group I or PAH,%		51.6%
PAH due to congenital heart disease, %		12.5%
PAH due to connective tissue disease, %		50%
PAH due to HIV, %		6.2%
PAH due to drugs and toxins, %		6.2%
PAH due to portal hypertension, %		6.2%
PAH idiopatic, %		18.7%
PH group II, %		9.7%
PH group III, %		19.3%
PH group IV (Chronic thromboembolic PH), %		9.7%
PH group V, %		9.7%
Arterial Hypertension, %		51%
Anemia, %		22%
COPD, %		24%
CKD, %		11%
ACE-inhibitors o ARBs, %		47%
B-blockers, %		41%
Digoxin, %		35%

**Table 2:** Differences between population’s subgroups (diagnosis of pulmonary hypertension not confirmed at right heart catheterization vs confirmed, pre-capillary vs post capillary pulmonary hypertension).

	Mean PH-16	Std.Dev.	Mean PH + 31	Std.Dev.	P	Mean post-PH 8	Std.Dev.	Mean pre-PH 23	Std.Dev.	P
Age (years)	60.1	9.5	61.8	12.3	n.s.	58,9	16.7	62.6	10.5	n.s.
Male (%)	44%		59%		n.s.	67%		55%		n.s.
BMI (kg/m2)	27.2	5.5	25.8	4.5	n.s.	26.2	4.8	25.6	4.5	n.s.
systolic pressure (mmHg)	121.3	17.9	117.7	19.5	n.s.	118.9	27.2	117.5	16.5	n.s.
heart rate (bpm)	77.4	13.1	79.8	11.7	n.s.	83.0	12.2	79.0	11.6	n.s.
Hypertension (%)	56%		44%		n.s.	56%		36%		n.s.
COPD (%)	19%		31%		n.s.	33%		27%		n.s.
Diabetes (%)	19%		22%		n.s.	22%		23%		n.s.
Chronic kidney disease (%)	6%		13%		n.s.	22%		9%		n.s.
NYHA class	2.3	0.5	2.8	0.5	<0.01	2.8	0.4	2.9	0.6	n.s.
										n.s.
VO2 AT (mL/min)	7.9	27.1	3.9	18.0	n.s.	11.8	33.4	0.7	0.2	n.s.
VO2 AT (mL/min/kg)	15.6	24.9	13.2	16.6	n.s.	19.9	30.5	10.4	3.1	n.s.
VO2 AT (%)	64.5	14.8	65.2	11.3	n.s.	68.6	14.5	62.3	7.2	n.s.
HR AT (bpm)	101.6	21.4	102.2	16.7	n.s.	111.0	18.7	98.6	15.2	n.s.
Watt AT (W)	33.9	14.9	24.5	11.5	<0.05	28.2	13.7	23.2	10.5	n.s.
Pulse AT (mL)	13.8	26.6	9.9	17.0	n.s.	16.6	31.6	7.1	1.6	n.s.
petCO2 AT (mmHg)	36.1	3.1	360	15.3	n.s.	33.2	6.7	33.3	7.8	n.s.
VO2 peak (ml/min)	0.9	0.3	4.1	18.0	n.s.	12.0	33.4	0.9	0.2	n.s.
VO2 peak (ml/min/Kg)	12.9	4.3	13.0	2.7	n.s.	12.7	2.8	13.2	2.8	n.s.
VO2 peak (%)	49.8	16.1	57.7	18.3	n.s.	50.7	17.7	60.7	18.6	n.s.
Watt peak (W)	62.6	26.9	40.9	18.0	<0.01	46.9	21.0	38.6	16.8	n.s.
Pulse peak (mL)	14.5	25.3	12.1	18.3	n.s.	17.7	31.3	9.8	9.5	n.s.
VO2 work slope (mL/min/W)	18.0	27.7	10.5	3.1	n.s.	8.0	2.1	11.5	2.9	<0.01
VT peak (L)	1.3	0.5	1.2	0.4	n.s.	1.2	0.4	1.2	0.5	n.s.
RR peak (1/min)	32.8	9.0	30.8	3.9	n.s.	32.5	5.2	30.0	2.9	n.s.
VE peak (L/min)	44.4	21.1	42.9	16.3	n.s.	47.3	17.7	41.6	15.9	n.s.
RQ peak	7.9	26.3	7.6	25.1	n.s.	12.4	33.6	5.8	21.8	n.s.
petCO2 peak (mmHg)	35.0	5.3	33.3	15.5	n.s.	31.6	8.2	30.7	9.4	n.s.
VE/VCO2 slope	31.3	5.2	41.5	17.1	<0.05	44.6	20.7	41.0	15.9	n.s.
Ventilatory power (mmHg)	3.9	0.7	3.2	1.2	n.s.	3.1	1.4	3.2	1.2	n.s.
Echocardiography										
LVEF (%)	55.0	7.1	51.6	9.2	n.s.	45.0	14.9	54.5	3.1	<0.01
PAsP (mmHg)	50.5	17.8	68.8	21.3	<0.01	68.9	28.5	68.2	18.8	n.s.
Right heart catheterization										
mPAP (mmHg)	17.3	4.7	39.1	10.8	<0.001	41.4	11.8	38.3	10.7	n.s.
PAPd (mmHg)	12.9	4.4	32.9	10.8	<0.001	33.0	13.0	32.9	10.3	n.s.
PCWP (mmHg)	8.5	4.4	12.6	6.8	<0.05	20.8	5.8	9.3	3.5	<0.001
Right atrial pressure (mmHg)	6.2	3.9	9.1	4.7	<0.05	12.8	3.1	7.8	4.5	<0.01
DPG (mmHg)	4.5	5.2	20.4	13.2	<0.001	11.6	16.4	23.6	10.5	<0.05
TPG (mmHg)	8.5	4.5	26.6	12.7	<0.001	20.7	14.5	29.0	11.4	n.s.
Cardiac output (L/min)	5.3	1.9	5.1	2.5	n.s.	5.4	4.3	5.0	1.4	n.s.
Cardiac index (L/min/m2)	3.3	0.9	3.1	1.7	n.s.	3.1	2.4	3.1	1.4	n.s.
RVP (WU)	1.7	0.9	6.1	4.1	<0.01	5.9	6.1	6.1	3.0	n.s.

After RHC, the patients were divided in two groups, 31 patients with PH (mPAP > 25 mmHg at RHC, PH + ) and 16 without PH (PH-). Between 31 patients with PH, 23 patients (74.2%) showed precapillary PH (PAWP ≤ 15 mmHg) and 8 patients (25.8%) postcapillary PH. According to last guidelines on hemodynamic classification of PH [1], 5 patients with combined post-capillary and pre-capillary PH (62.5%) and 3 patients with isolated postcapillary PH (37.5%) were identified.

Among PH + patients, 16 belonged to ESC guidelines group I (51.61%) and 6 to group III (19.35%), while the remaining were distributed into the others groups (Table 1).

**Fig. 1:**
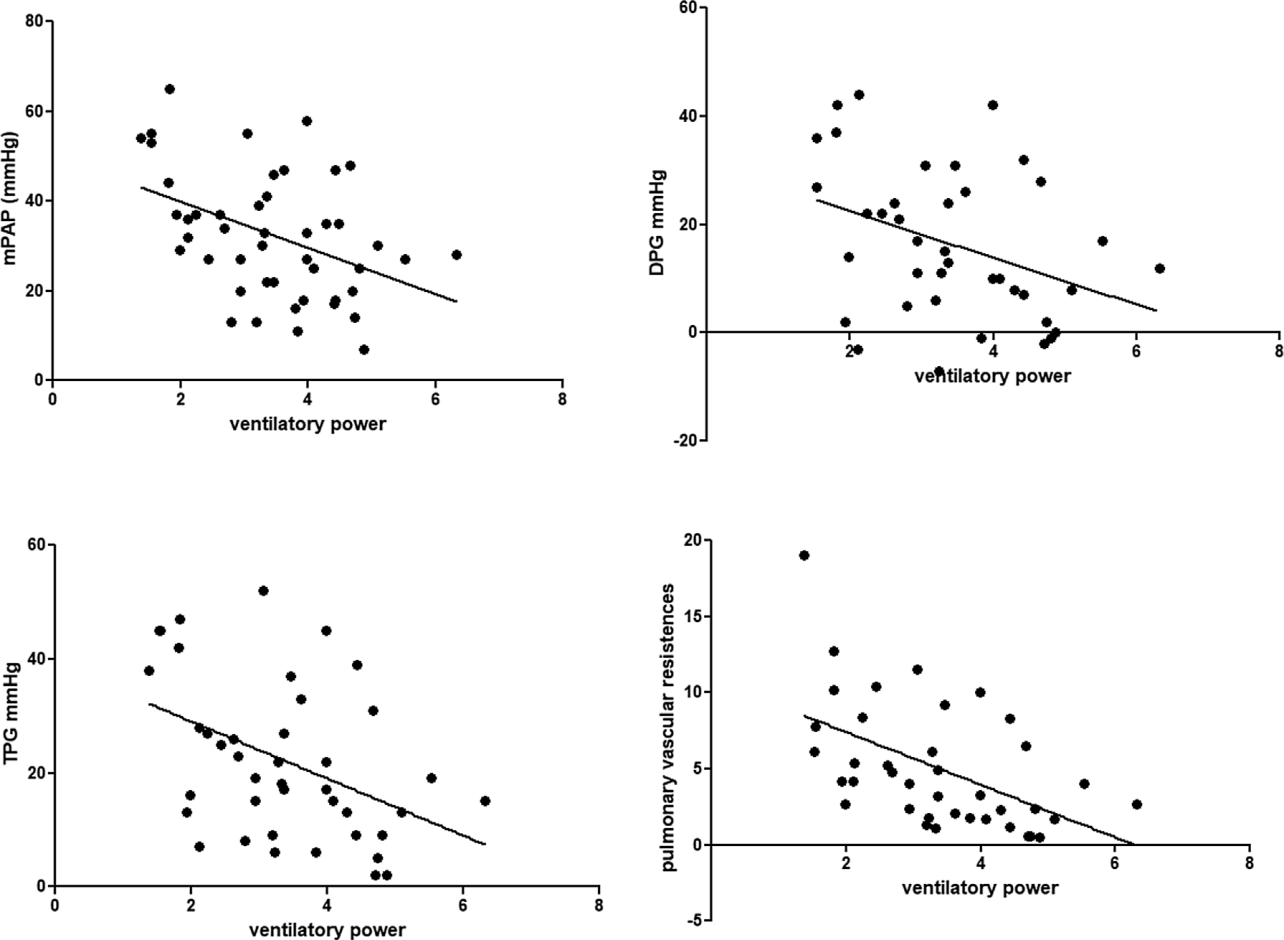
Correlations between Ventilatory Power at cardiopulmonary exercise test and hemodynamic parameters assessed at right heart catheterization.

VP values assessed at CPET were inversely correlated with the following hemodynamic parameters at RHC: mPAP (r −0.427, p 0.003), diastolic pressure gradient (DPG, r −0.36, p 0.019), trans-pulmonary pressure gradient (TPG, r −0.43, p 0.004), pulmonary vascular resistance (PVR, r −0.52, p 0.001) (Fig. 1). Correlations remained significant even after correction at multivariate analysis including age and gender (p < 0.01 for mPAP, DPG, and TPG,<0.001 for PVR).

VP values below median (3.4 mmHg) identified subjects with mPAP ≥ 25 mmHg with an OR of 4.5 (95% confidence interval 1.05–19.36, p < 0.05), an accuracy of 0.712 at ROC curve analysis (95% confidence interval 0.534–0.852, p < 0.05) and a positive predictive power of 82%.

**Fig. 2:**
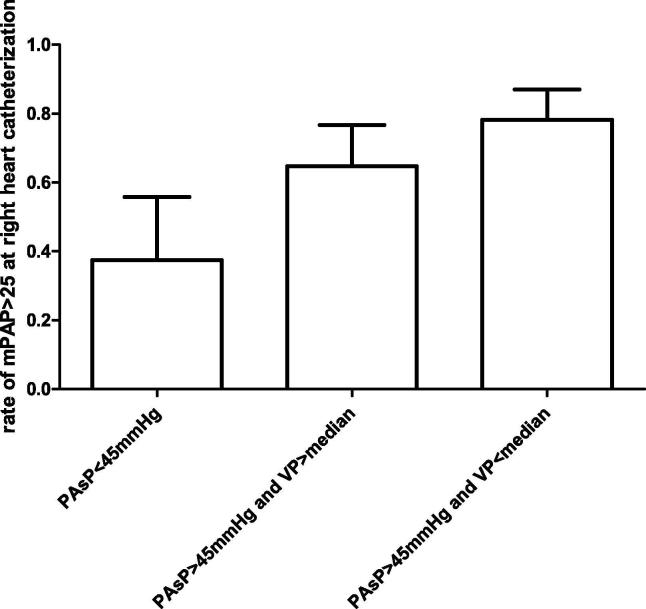
Confirmed diagnosis of pulmonary hypertension rates at right heart catheterization with systolic PAP < 45 mmHg at echocardiography Doppler examination, systolic PAP > 45 mmHg and ventilatory power values above median and below median (p for trend < 0.001).

Confirmed diagnosis of pH at RHC rates were progressively high in case of PAsP values > 45 mmHg at Doppler echocardiography and both both PAsP values > 45 mmHg and VP values < median (38%, 65%, and 78% respectively, p for trend 0.001, OR 30 vs controls with PAsP < 45 mmHg, p < 0.01, Fig. 2).

## 4 Discussion

In this paper we show for the first time significant correlations between VP and PAH parameters at RHC. In patients with suspect of PAH, VP might provide useful information to predict results at RHC.

Currently, the suspect of pH is based on a limited number of echocardiographic parameters such as PAsP, poorly able, however, to discriminate pre-capillary from post-capillary PH. RHC is therefore usually required to confirm the diagnosis of pre-capillary PH.

In this study, VP values assessed at CPET were inversely correlated with several hemodynamic parameters at RHC: mPAP, DPG, TPG, PVR.VP values below median identified subjects with PH with acceptable levels of accuracy and positive predictive power.

These results appear particularly interesting for a possible selection of patients who should be assessed at RHC to confirm the suspected diagnosis of PH.

Some CPET parameters have been correlated with hemodynamic findings: peak VO_2_ may be inversely correlated with mPAP [8]. Nishio et al. demonstrated that peak VO_2_ correlates with PVR in PAH patients but not in chronic HF patients [9]. Furthermore, peak VO_2_ correlates with PAWP for chronic heart failure patients but not for PAH patients, while the VE/VCO_2_ slope correlates with PAWP but not with PVR for chronic heart failure patients. Very recently Dumitrescu et al. demonstrated in patients with systemic sclerosis that PeakVO_2_ and VE/VCO_2_ had a high correlation with PAP, TPG and PVR [10].

Less, however, is known about the correlations between CPET and hemodynamic parameters at RHC indicative of PH. Recently we demonstrated significant correlations between novel CPET parameters, the intercept of ventilation (VEint) on the VE vs. carbon dioxide production (VCO2) and PH at RHC. Correlations between VE/VCO2, mPAP and DPG may be helpful in discriminating patients with isolated post-capillary PH from others with combined post-capillary and pre-capillary PH [11].

Hirashiki et al. [7] used VP to evaluate modifications in exercise capacity over time under ‘‘goal-oriented’’ sequential combination therapy in patients newly diagnosed with PAH. VP increased after three months of treatment and still at six months. At the same time, the PAH patients showed higher peak VO2 and peak SBP values, lower VE/VCO2 slope after six months; both mPAP and PVR were decreased after 12 months. Such results are in line with our data, showing that VP increases inversely than mPAP and PVR.

Furthermore, in this study we also demonstrated inverse correlations between VP and other hemodynamic parameters, such as DPG and PVR. Such correlations may be helpful in discriminating patients with isolated post-capillary PH from others with combined post-capillary and pre-capillary PH.

In previous ESC guidelines (2009) [12], TPG values were discriminant for “passive” post-capillary PH (mPAP ≥ 25 mmHg, PAWP > 15 mmHg and TPG < 12 mmHg) from “reactive” post-capillary PH (mPAP ≥ 25 mmHg, PAWP > 15 mmHg and TPG ≥ 12 mmHg); TPG is influenced by all the determinants of mPAP, including flow, resistance and left heart filling pressure. Naeije et al. [13] demonstrated that DPG is superior to TPG for the diagnosis of “out of proportion” PH. Compared to TPG, DPG may be a more sensitive and specific indicator for PH due to left heart disease with significant pulmonary vascular disease [14].

In latest ESC guidelines [1] the combined use of DPG and PVR is recommended to define different types of PH-left heart disease (Ipc-PH and Cpc-PH). From a clinical perspective, that is extremely relevant since the vasodilatation of pH specific drugs may be useful in case of vasoreactivity, or, furthermore, of precapillary problems as in the Cpc-PH.

Non invasive methods could be therefore useful in order to distinguish precapillary PH from postcapillary PH, and mainly, Cpc-PH from Ipc-PH.

In our study increasing rates of subjects with mPAP values > 25 mmHg at RHC were found in subjects with PAsP values > 45 mmHg at Doppler echocardiography and VP values below median. The capacity of non invasive methods to predict invasive confirmation of pH is still matter of debate [15]. Echocardiography and CPET were used in association by Guazzi et al. to predict the outcome of heart failure patients [16]; Badagliacca et al., instead, used this non-invasive methods to predict the outcome in idiopathic PAH [17]. Zhao et al. applied CPET to improve the specificity of echocardiography in patients with suspected PH [18], while Held et al. used CPET to detect chronic thromboembolic PH in patients with normal echocardiography [19]. According to our findings, a careful assessment of non invasive testing may be extremely useful in identifying those subjects with an increased probability of higher DPG levels at RHC. Such preliminary data, however, deserve further confirmation in larger populations.

## 5 Conclusions

In patients with suspected PAH, VP assessed at CPET might provide further information in predicting PAH at RHC. Correlations with PVR and DPG may be helpful in differentiating patients with isolated post-capillary PH from those with combined post-capillary and pre-capillary.

## 6 Limitations

Main limitation of the study is the relatively small number of patients enrolled; these preliminary results need to be confirmed as “proof of concept” in larger cohorts of patients and in a properly powered multicentric study.

## CRediT authorship contribution statement

**Michele Correale:** Conceptualization, Methodology, Investigation, Data curation, Writing - original draft, Project administration. **Ilenia Monaco:** Investigation, Data curation. **Armando Ferraretti:** Conceptualization, Methodology, Investigation, Data curation. **Lucia Tricarico:** Methodology, Investigation, Data curation. **Monica Sicuranza:** Investigation, Data curation. **Anna Maria Gallotta:** Investigation, Data curation. **Ennio Sascia Formica:** Investigation, Data curation. **Gianfranco Acanfora:** Investigation, Data curation. **Matteo Di Biase:** Supervision. **Natale Daniele Brunetti:** Conceptualization, Methodology, Investigation, Data curation, Writing - review & editing, Supervision, Visualization, Project administration, Formal analysis.

## Declaration of Competing Interest

The authors declare that they have no known competing financial interests or personal relationships that could have appeared to influence the work reported in this paper.
